# Vascular cognitive impairment in *Pemphigus vulgaris*: A case report

**DOI:** 10.1590/S1980-57642012DN06030014

**Published:** 2012

**Authors:** José Ibiapina Siqueira-Neto, Paulo Marcelo Gondim Sales, Emmanuelle Silva Tavares Sobreira, Aline Miranda Limeira, Heline Bessa Araujo, Júnia Vieira dos Santos, José Daniel Vieira de Castro

**Affiliations:** 1 MD, PhD, Associate Professor of Neurology in the Clinical Medicine Department of Federal University of Ceará, Fortaleza CE, Brazil.; 2 Medical Student, Walter Cantidio University Hospital, Federal University of Ceara, Fortaleza CE, Brazil.; 3 MSc, Psychologist, Behavioral Neurology Group of Federal University of Ceara, Fortaleza CE, Brazil.; 4 MD, Medical Resident of Neurology, Walter Cantidio University Hospital, Federal University of Ceara, Fortaleza CE, Brazil.; 5 MD, Medical Resident of Neurology, Walter Cantidio University Hospital, Federal University of Ceara, Fortaleza CE, Brazil.; 6 Medical Student, Walter Cantidio University Hospital, Federal University of Ceara, Fortaleza CE, Brazil.; 7 MD, PhD, Adjunct Professor of Neuroradiology in the Clinical Medicine Department of Federal University of Ceará, Fortaleza CE, Brazil..

**Keywords:** *Pemphigus vulgaris*, cognitive vascular impairment, neuropsychological testing, auto-immune diseases, dementia

## Abstract

*Pemphigus vulgaris* is a systemic auto-immune medical condition that mainly manifests with changes in skin and vasculopathy. This is a case report of a 69-year-old male with confirmed histopathologic diagnosis of *Pemphigus vulgaris* presenting ulterior Cognitive Impairment, mostly in executive function. The patient was treated using steroids, immunomodulatory therapy, fluoxetine and galantamine. Neuropsychological testing and magnetic resonance (MRI) were performed. This is the first report of correlational cognitive impairment with *Pemphigus vulgaris* in the literature. Physicians should be aware of vascular causes for cognitive impairment in patients presenting auto-immune conditions.

## Abstract (pt)

*Phemphigus vulgaris* é uma condição médica sistêmica autoimune que principalmente se manifesta com alterações de pele e vasculopatia. Este é um caso de um homem de 69 anos com diagnóstico histopatológico de *Phemphigus vulgaris* apresentando posterior comprometimento cognitivo, predominante em funções executivas. O paciente foi tratado com esteroides, terapia imunomoduladora, fluoxetina e galantamina. Avaliação neuropsicológica e ressonância magnética foram realizados. Este é o primeiro relato correlacionando comprometimento cognitivo a pênfigo vulgar na literatura. Os clínicos devem estar cientes das causas vasculares para comprometimento cognitivo em pacientes se apresentando com condições autoimunes.

## INTRODUCTION

Vascular Cognitive Impairment (VCI) and Vascular Dementia (VaD) are often responsible for cognitive complaints in memory clinics. Traditionally, vascular impairment is considered the second most common etiology of dementia after Alzheimer's Disease (AD).^1^ For instance, the prevalence rate of Post-Stroke Dementia - one of the most frequent subtypes of VaD - varies from 12.2% to 31.8% within 3 months to 1 year after the initial stroke,^2^ but the magnitude of VaD remains uncertain and largely dependent on complementary investigation, especially using neuroimaging tools. Most of the studies available estimate the burden of VaD at around 20 to 30% of all cases of dementia.^1^

For clinical purposes, we have considered three large groups of VaD: post-stroke dementia, subcortical vascular dementia and mixed dementia (AD + VaD).^2-4^ In post-stroke dementia, we further delineated three subgroups: strategic infarcts, hemorrhagic dementia and hypoperfusion dementia.

The causes of VCI are multiple. Some less commonly observed disorders include CADASIL, Binswanger's, Connective Tissue Disorders and Amyloid Angiopathy.^3,4^ *Pemphigus vulgaris* was hitherto not listed as a cause of cognitive impairment, despite the fact that there is a large list of autoimmune disorders linked to cognitive complaints.^5^ We present the case of a patient diagnosed with *Pemphigus vulgaris* that developed cognitive complaints before 65 years without previous focal signs or any evidence of other predisposing factors, except very mild hypertension. He developed small-vessel vascular dementia and fulfilled the NINDS-AIREN criteria for VaD.^6^

## CASE REPORT

Our patient is a 69-year-old male with 5 years of formal education, memory complaints and *Pemphigus vulgaris*. No previous stroke episodes were reported and he presented no focal neurological signs. His blood pressure was strictly controlled during the follow-up and no hypertensive crises were reported. No tobacco or alcohol abuse was reported and the family history was negative for VaD. No additional risk factors for VaD were present, except mild hypertension controlled with hydrochlorothiazide (12.5 mg PO) and captopril (25 mg BID).

The skin lesions compatible with pemphigus (vesiculobullous dermatitis associated with epidermolysis) were first noticed in 1998. A total of 4 biopsies were performed, all of which were consistent with the diagnosed of pemphigus vulgaris. From April 1998 to December 2005 he was treated using immunosuppressive therapy consisting of prednisone (40 mg PO) associated with dapsone (100 mg PO) or azathioprine (150 mg PO), occasionally relapsing during attempts to reduce the dose of prednisone. He also developed opportunistic infections that required hospitalization for intravenous antibiotic therapy. No major changes in glycemic level, lipid profile or blood pressure were reported during these episodes, while image investigation revealed no suggestive signs of vasculitis in the CNS.

In 2006, the first behavioral changes were documented: apathy, emotional lability and melancholic mood. Fluoxetine 20 mg once a day was started with a good mood response 4 weeks later. However, during the follow-up consultations, the Psychiatric team readjusted the dose to 20 mg BID, due to great apathy, loss of initiative and occasional hallucinations. Due to complaints about loss of short-term memory, he was sent to our memory clinic and the first MMSE performed in 2008 yielded a score of 29/30. A standardized protocol to investigate memory complaints was performed initially (complete blood count, biochemistry, B_12_, folic acid, thyroid function) and no significant changes were observed.

Since the patient presented no signs of impairment in activities of daily living and no quantifiable loss in short-term memory, a diagnosis of non-amnestic Mild Cognitive Impairment was suggested. During the follow-up evaluation in 2009, evidence of significant cognitive impairment was observed, including an MMSE score of 23/30, with the loss of one point in praxis and the clock-drawing test 03/05. Verbal Fluency (VF) was 11 animals per minute. In 2010 and 2011 the patient persisted with borderline changes on routine trail cognitive tests (MMSE and VF), but progressive impairment in activities of daily living developed, culminating in the need for constant supervision during the execution and planning of all daily tasks.

We performed an investigation to assess possible etiologies. Blood tests (results of 08/2012): serum creatinine (0.9 mg/dL), total cholesterol (176 mg/dL), serum glucose (83 mg/dL), LDL cholesterol (82 mg/dL), HDL cholesterol (76 mg/dL), VLDL cholesterol (21 mg/dL), triglycerides(105 mg/dL), AST (26 u), ALT (18 u), rheumatoid factor (7.10 u; reference value <14), VDRL negative (flocculation), protein electrophoresis (total protein: 6.40; albumin/gama globulin relation: 1.94, with the absence of anomalous fraction), anti-hepatitis C virus negative, anti-nuclear antibodies negative, HbA_1_C (3.8%). We also collected blood samples for other autoimmune conditions, complement fractions, PCR for Lyme disease, antiphospholipid antibodies, Lupus anticoagulant, and cryoglobulins but the results are not yet available.

**Figure 1:**
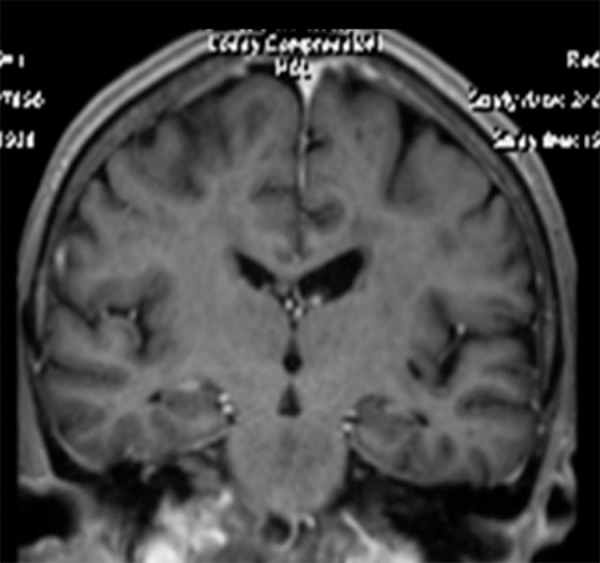
Coronal MRI presenting mesial hippocampal preservation.

**Figure 2:**
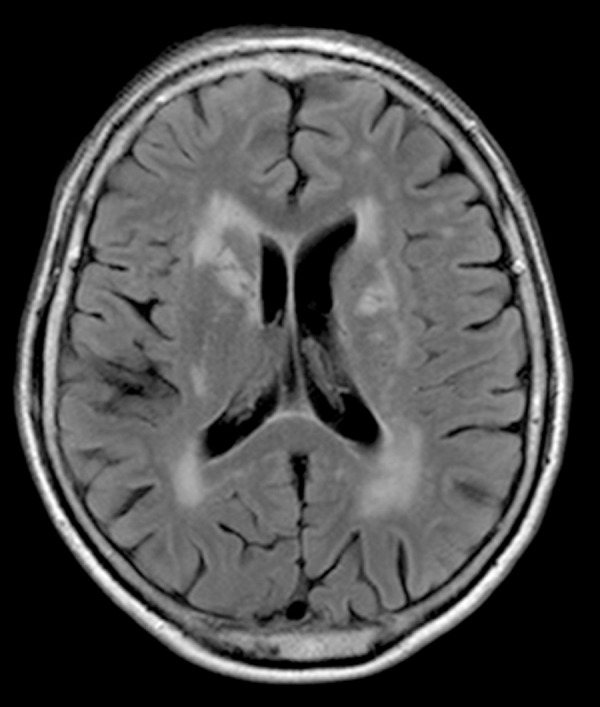
Flair MRI revealing moderate-severe lesions in white matter.

The duplex scan of the carotid showed no atheromatosis, hyperplasia in miointimal complex, or changes in arterial blood flow. The MRI revealed lacunar infarcts in the thalamus and left hemisphere capsular nuclei, intense white matter changes with symmetric hippocampus (normal volume and signal), plus mild cortical-subcortical changes (Figure 1 and 2). Another MRI was done in 2009 but the films were not available for comparison. Intense white matter changes were described. The MRI Angiogram showed normal blood flow and no evidence of cranial atheromatosis. Both the holter study and echocardiogram were normal.

As the neuroimaging results suggested, we have a case of subcortical dementia due to small vessel impairment. This case was not associated with common risk factors usually present in this type of dementia. Galantamine was initiated up to a 24 mg/day dose. The patient did not use statins. Hypertension was easy controlled with the same anti-hypertensive combination as mentioned, *plus* aspirin (100 mg PO). During the last consultation in May of 2012, MMSE performance stabilized at 24/30 and the patient's wife reported great improvement in her husband on task-solving situations at home, and that he was now helping in domestic work and presenting a regular mood, having only fluctuating short-term memory deficits.

A neuropsychological (NP) evaluation was performed, showing deficits in executive function and praxis with some degree of perseveration in short-term memory Table 1).

**Table 1:** Neuropsychological Cognitive Battery.

Cognitive domain		Values obtained	Percentile	Final classification	Cut-off
Intelligence	Estimated intelligence quotient (vocabulary + cubes)	17	89	Below average	-
Working memory	Digits - WAIS III	9	37	Average	-
Episodic memory (verbal/visual)	RAVLT - Total	26	≤ 0.1*	Deficitary	-
	RAVLT - B (interference list)	0	≤ 0.1*	Deficitary	-
	RAVLT - VI (evocation after interference)	7	17	Below average	-
	RAVLT - Long term memory	4	≤ 0.1*	Deficitary	-
	RAVLT - recognition	4	4	Borderline	-
	Rey's Complex Figure Test - Evocation	6.5	18	Below average	-
	Rey's Complex Figure Test - Evocation (time)	11 minutes	≤0.1*	Deficitary	-
Constructive praxis	Rey's Complex Figure Test - Copy	11.5	≤0.1*	Deficitary	-
	Rey's Complex Figure Test - Copy time	21 minutes	≤0.1*	Deficitary	-
Language	Boston Naming Test	37	32	Below average	-
Executive Function / Attention	FAS	16	20	Below average	-
	Color Trails 1	377 seconds	≤0.1*	Deficitary	-
	Color Trails 2	638 seconds	≤0.1*	Deficitary	-
	Stroop Test I	33 seconds	≤0.1*	Deficitary	-
	Number of mistakes	0			
	Stroop Test II	58 seconds	≤0.1*	Deficitary	-
	Number of Mistakes	4			-
	Stroop Test III	35 seconds	33	Average	-
	Number of Mistakes	0			
Praxis	Clock Drawing Test	6	-	-	<11
Affect and mood	Hospital Anxiety and Depression Scale				
	Depression	1	-	-	9
	Anxiety	5	-	-	8
Activities of daily living	Functional Activities Questionnaire	6	-	-	>5
DRS (Mattis)	Global	113	-	-	<123
	Attention	33	-	-	<36
	Initiative/Perseveration	25	-	-	<33
	Construction	3	-	-	<6
	Conceptualization	28	-	-	<31
	Memory	24	-	-	<17

* Abnormal values; DRS: Dementia Rate Scale; FAS: Phonemic Verbal Fluency; RAVLT: Rey Auditory Verbal Learning Test; WAIS: Wechsler Adult Intelligence Scale.

## DISCUSSION

Many autoimmune mechanisms can induce changes in the vascular network of the central nervous system (CNS), leading to ulterior VCI, such as arteritis of large vessels. The association between *Pemphigus vulgaris* and VaD, with subcortical impairment in small vessels, was not documented in the literature. Isolated vasculitis of the CNS and diseases of the connective tissue were described as possible etiologies of vascular dementia,^5,7,9^ but only a few cases were described.

No references suggesting a possible correlation between *Pemphigus vulgaris* and VCI were reported in the literature.^7-9^ *Pemphigus vulgaris* leads to changes in skin, mucosal surfaces and sometimes even lung in its paraneoplastic form,^10^ indicating a likely role of autoimmune mechanisms in this phenomenon.^11^ The diagnosis is established with skin biopsies and methods for the detection of auto-antibodies.^12^

Our patient did not have risk factors commonly observed in patients with VCI as we documented. Only mild hypertension, easily controlled with an association of two drugs at small doses was reported, which would be incompatible with the degree of white matter changes observed. The clinical course was typical of small vessel disease. Some limitations should be taken account in the interpretation of this data, such as the absence of a cerebrospinal fluid study, since we did not have evidence of CNS vasculitis that would justify this investigation, such as decreased level of consciousness or confusion, focal neurologic deficits and any hospitalization due to stroke-like episodes. For the same reason, we did not perform angiography studies, since the MRI angiogram disclosed no evidence of large vessel arteritis. PET CT was not available in our service and cerebral biopsy was not performed.

VCI is growing in burden and prevalence, probably representing better diagnostic training of the health care team and imaging tests. It is quite possible that this burden can grow even further, if other specialists become aware that other non-usual vascular etiologies may be responsible, or at least co-responsible, for cognitive impairment.

The progression of *Pemphigus vulgaris* is variable and includes relapses that prompt the need for constant changes in the immunosuppressive treatment. Many patients need long-standing corticosteroid therapy. In a Tunisian prospective study, fulminant cases that led to death were reported,^13^ but none of the previously reported cases suggest a possible small-vessel disease etiology with slow progression course.

In conclusion, *Pemphigus vulgaris* is a skin disease with a variable prognosis that involves auto-immune mechanisms. Many aspects of the disease are complex and still unclear. There is an interface between *Pemphigus vulgaris* and diseases of the connective tissue, eventually leading to CNS vasculitis of both large and small vessels. It is an issue that requires further elucidation with bigger cohort studies.

We reported a patient with confirmed *Pemphigus vulgaris* that evolved with progressive global cognitive impairment compatible with subcortical dementia characterized predominantly by executive dysfunction, moderate-severe white matter changes on MRI and absence of commonly related risk factors of VaD. Galantamine seemed to have good efficacy, especially concerning improved activities of daily living.
